# Cord blood ceramides facilitate early risk identification into childhood metabolic health

**DOI:** 10.1093/nsr/nwae352

**Published:** 2024-10-03

**Authors:** Jia Zheng, Sin Man Lam, Binhua Jiang, Lili Mao, Jieying Liu, Qian Zhang, Miao Yu, Wei Ling Florence Lim, Claudia H T Tam, William L Lowe, Wing Hung Tam, Ying Gao, Junqing Zhang, Ronald C W Ma, Xinhua Xiao, Guanghou Shui

**Affiliations:** Department of Endocrinology, Peking University First Hospital, China; Key Laboratory of Endocrinology of National Health Commission, Diabetes Research Center of Chinese Academy of Medical Sciences, Department of Endocrinology, Peking Union Medical College Hospital, Peking Union Medical College, Chinese Academy of Medical Sciences, China; State Key Laboratory of Molecular Developmental Biology, Institute of Genetics and Developmental Biology, Chinese Academy of Sciences, China; LipidALL Technologies Company Limited, China; LipidALL Technologies Company Limited, China; Key Laboratory of Endocrinology of National Health Commission, Diabetes Research Center of Chinese Academy of Medical Sciences, Department of Endocrinology, Peking Union Medical College Hospital, Peking Union Medical College, Chinese Academy of Medical Sciences, China; Key Laboratory of Endocrinology of National Health Commission, Diabetes Research Center of Chinese Academy of Medical Sciences, Department of Endocrinology, Peking Union Medical College Hospital, Peking Union Medical College, Chinese Academy of Medical Sciences, China; Key Laboratory of Endocrinology of National Health Commission, Diabetes Research Center of Chinese Academy of Medical Sciences, Department of Endocrinology, Peking Union Medical College Hospital, Peking Union Medical College, Chinese Academy of Medical Sciences, China; Key Laboratory of Endocrinology of National Health Commission, Diabetes Research Center of Chinese Academy of Medical Sciences, Department of Endocrinology, Peking Union Medical College Hospital, Peking Union Medical College, Chinese Academy of Medical Sciences, China; LipidALL Technologies Company Limited, China; Department of Medicine and Therapeutics; Hong Kong Institute of Diabetes and Obesity, Li Ka Shing Institute of Health Sciences, The Chinese University of Hong Kong, China; Northwestern University Feinberg School of Medicine, USA; Department of Obstetrics and Gynaecology, The Chinese University of Hong Kong, China; Department of Endocrinology, Peking University First Hospital, China; Department of Endocrinology, Peking University First Hospital, China; Department of Medicine and Therapeutics; Hong Kong Institute of Diabetes and Obesity, Li Ka Shing Institute of Health Sciences, The Chinese University of Hong Kong, China; Key Laboratory of Endocrinology of National Health Commission, Diabetes Research Center of Chinese Academy of Medical Sciences, Department of Endocrinology, Peking Union Medical College Hospital, Peking Union Medical College, Chinese Academy of Medical Sciences, China; State Key Laboratory of Molecular Developmental Biology, Institute of Genetics and Developmental Biology, Chinese Academy of Sciences, China; University of Chinese Academy of Sciences, China

The Developmental Origins of Health and Disease (DOHaD) theory highlights the impact of intrauterine exposures commencing from pre-conception that significantly affect health trajectories throughout later life [1]. Our previous findings indicated that low birth weight (<3000 g) is an independent risk factor for impaired glucose regulation and type 2 diabetes (T2D) at a later life stage [2]. Molecular mechanisms underlying the risks of non-optimal birth weights leading to metabolic disturbances later in life, however, are not yet fully understood. Lipids represent a highly diverse class of biological molecules underpinning various physiological functions, including cellular signaling crucial to fetal growth and development. Previous studies examining maternal and fetal links had predominantly focused on pregnancy outcomes at birth [3,4]. A pilot study reporting associations between maternal and cord blood lipidomes suffered from a small sample size and did not examine the impact of lipid disturbances at birth on future childhood metabolism [5]. Along this line, sphingolipids elicit important functions in both development and post-development stages [6,7].

In this study, we analyzed sphingolipidome using targeted and quantitative approaches [8,9] of umbilical cord blood from 152 mother-offspring dyads to delineate functional sphingolipids that were associated with birth weight. Proteomics was also performed on a subset of placental tissues. Trans-omics integration of cord blood lipidomics with placental proteomics was leveraged to elucidate the mechanistic function of cord blood sphingolipids that underlie differences in infant birth weight. Finally, the associations of cord blood sphingolipids with childhood metabolic health at 7 years were investigated in an independent cohort.

Cord blood sphingolipid perturbations associated with birth weight deviations away from the optimal level were examined, with pairwise comparisons made using normal birth weight (NBW, birth weight ≥3000 g and <4000 g) as the reference group. Limma modelling uncovered sphingolipid perturbations in cord blood of infants from the low birth weight (LBW, birth weight <3000 g) and high birth weight (HBW, birth weight ≥4000 g) groups, with respect to the reference NBW group (Fig. 1A, Fig. S1A). Distinct patterns of lipid changes were observed amongst major sphingolipid classes (Fig. 1B, Fig. S1B). In particular, cord blood ceramides (Cer) with medium to very long fatty acyl chains (C20-C26) exhibited V-pattern of changes with deviations from optimal birth weights (Fig. 1A and B), implying that higher than optimal levels of these cord blood lipids were associated with unhealthy birth weights. Very long-chain (C22-C26) Cer (VLC-Cer) were significantly elevated in the cord blood of HBW compared to NBW, albeit no significant differences in protein levels of placental ceramide synthase 2 (CerS2) (Fig. S1C), which is responsible for the biosynthesis of VLC-Cer [10], were observed across the three groups. Cord blood level of VLC-Cer such as Cer d18:0/24:1, was positively and significantly correlated (*r* = 0.536) with endogenous level in maternal plasma (Fig. S1D), indicating that increased cord blood VLC-Cer in HBW and LBW might stem from maternal blood supply instead of *de novo* biosynthesis within the placenta. On another note, sulfatides (SLs) displayed an inverted V-pattern while sphingomyelins (SMs) were distinctly reduced in HBW compared to NBW and LBW (Fig. S1A and B).

**Figure 1.**
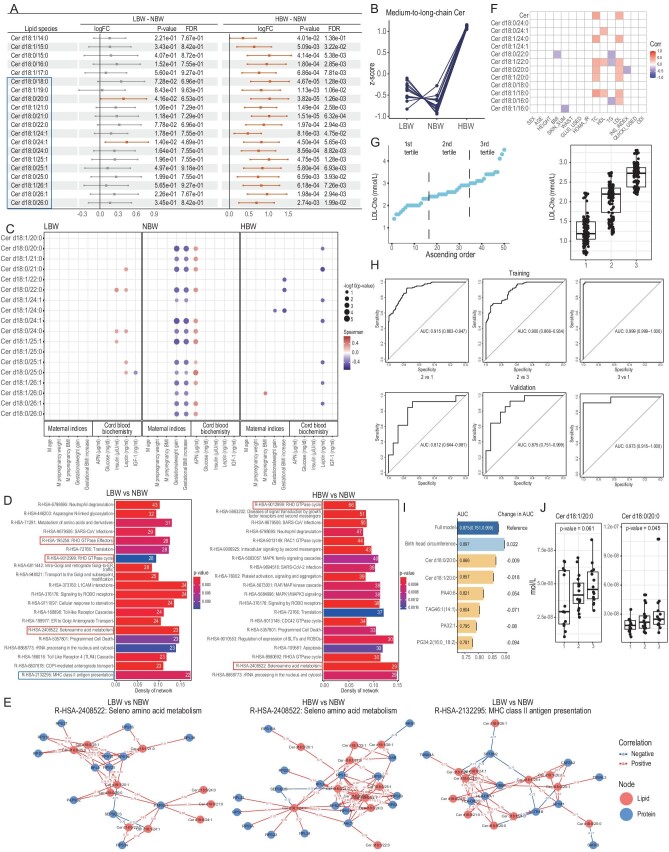
Association between cord blood Cer and offspring metabolic health. (A) Cord blood Cer were associated with infant birth weights. Forest plot illustrates differential lipids obtained using the limma model for LBW relative to NBW (LBW-NBW) and HBW relative to NBW (HBW-NBW). Log2 fold change (95% CI), *P* value, and false discovery rate (FDR, 0.05; Benjamini–Hochberg method) are presented. Blue rectangle contains Cer with V-pattern of changes from LBW through NBW to HBW. Red dots indicate Cer significantly upregulated relative to NBW, while grey dots denote non-significant changes. Lipid species are indicated on the y axis. (B) V-pattern of changes in medium-to-long chain cord blood Cer across different birth weight groups illustrated as line plots drawn using *z*-scores. (C) Correlation between cord blood Cer and clinical indices. Bubble plots illustrate the Spearman correlations between cord blood lipids and maternal indices and other indicators relevant to cord blood biochemistry in LBW, NBW and HBW groups. Only statistically significant correlations are shown. Size of the bubble represents magnitude of *P* values from Spearman correlations, with positive correlations in red and negative correlations in blue. Color intensity denotes the strength of correlation. (D) Trans-omics integration revealed molecular pathways associated with changes in cord blood very long-chain (C20-C26) Cer. Over-representation analysis (ORA) of pathways using the Reactome database based on placental proteins correlated (*P* <0.05, |r|>0.7) with very long-chain (C20-C26) Cer in LBW + NBW, and HBW + NBW, respectively. Significant pathways (*P* <0.05) were ranked based on density of networks. Pathways boxed in red denote common pathways associated with cord blood Cer levels for both LBW + NBW and HBW + NBW analyses, while the pathway boxed in blue was distinct to LBW + NBW analysis. (E) Correlation networks of proteins and lipids categorized under the Reactome pathway R-HSA-2408522 seleno amino acid metabolism for the LBW + NBW and HBW + NBW groups, respectively. SEPSECS, a key enzyme in the biosynthesis of selenoproteins, was negatively correlated to several very long-chain Cer for both LBW and NBW. Correlation networks of proteins and lipids categorized under the Reactome pathway R-HSA-2132295 MHC class II antigen for the LBW + NBW group. Red and blue numerals on edges indicate positive and negative correlation coefficients, respectively; red nodes denote lipids and blue nodes denote proteins. (F) Correlation analysis of umbilical cord blood Cer with clinical parameters. Spearman correlation coefficients were calculated and shown in a heatmap plot. Significant correlations were indicated by color, with positive correlations in red and negative correlations in blue. (G) Baseline levels of cord blood Cer at birth predict childhood lipid metabolism at the 7th year. LDL-Cho at the 7th year were divided into tertiles, labeled as groups 1, 2 and 3. (H) ROC curves on model performance of lasso-selected variables in discriminating different tertiles of LDL-Cho at the 7th year. The cohort was randomly divided into training (90%) and validation (10%) sets. Ten-fold cross validation was conducted and ROC curves were created using the combined training and validation samples. (I) Corresponding changes in AUC of ROC curves distinguishing participants in the 3rd from the 2nd tertiles based on validation set when removing variables one by one. (J) Boxplots showed changes in cord blood Cer d18:1/20:0 and Cer d18:0/20:0 across the three tertiles. *P* values were from ANOVA test for linear regression analysis. NBW, normal birth weight; LBW, low birth weight; HBW, high birth weight; Cer, ceramides; APN, adiponectin; IGF-1, insulin-like growth factor 1; BMI, body mass index; SF: skinfold thickness; WC: waist circumference; Glu0: fasting glucose; HOMA-IR, homeostatic model assessment for insulin resistance; TC, total cholesterol; HDL-Cho, high density lipoprotein cholesterol; TG, triglycerides; LDL-Cho, low density lipoprotein cholesterol; INS_index, insulinogenic index; QUICKI, quantitative insulin sensitivity check index; ODI: oral disposition index; AUC, area under the curve; ROC, receiver operating characteristic curve.

We next examined the correlations between these functional cord blood sphingolipids with various maternal indices and measures of cord blood microenvironment (Fig. 1C, Fig. S1E, Table S1). Correlations between long chain SLs with maternal age and cord blood insulin level were observed predominantly in HBW (Fig. S1E). SL reductions in HBW (Fig. S1A and B) were correlated with elevated cord blood insulin (Fig. S1E), suggesting perturbed glucose regulation commonly observed in fetal macrosomia, which can significantly increase the risk of childhood obesity and metabolic syndrome later in life [11]. Cord blood Cer was positively correlated with adiponectin (APN) specifically in NBW. APN signalling is generally associated with improved metabolic health, including enhanced insulin sensitivity and lowered inflammation. As APN receptors possess basal ceramidase activity, receptor binding and subsequent activation may serve to keep cord blood Cer at low levels in the NBW group. In groups with non-optimal birth weights, however, APN-mediated suppression of endogenous Cer levels was probably lost. Cord blood Cer was negatively correlated with leptin in HBW, but positively correlated with leptin in LBW. The differing correlations between cord blood Cer and leptin levels might be ascribed to differences in leptin sensitivity in the two birth weight groups [12,13]. SMs did not elicit any obvious correlation with maternal indices and indicators of cord blood biochemistry (Fig. S1E).

To systematically probe the function of cord blood Cer in regulating birth weight, we performed trans-omics integration using paired sets of placental proteome and cord blood Cer data. We conducted integrated analyses separately for LBW and NBW (*n* = 10), as well as HBW and NBW (*n* = 10), to elucidate perturbed molecular pathways as birth weight deviates bi-directionally from the optimum. ORA analysis using the Reactome database based on significantly correlated proteins uncovered pathways associated with perturbations in cord blood Cer levels (Fig. 1D). The ‘Seleno amino acid metabolism’ emerged as the top common significant pathway (for both LBW and HBW) as birth weight diverges from the optimum (Fig. 1D). Lipid-protein correlation networks revealed that VLC-Cer (C22-C25) were negatively correlated with the protein level of O-phosphoseryl-tRNA(Sec) selenium transferase (SEPSECS), while positively correlated with numerous components of the small and large ribosomal subunit proteins (RPS, RPL) (Fig. 1E). SEPSECS converts O-phosphoseryl-tRNA(Sec) to selenocysteinyl-tRNA(Sec) to generate the unique tRNA specific to selenoprotein production. Indeed, the levels of both selenoprotein F (SELENOF) and selenoprotein K (SELENOK) were particularly reduced in the placenta tissues of HBW relative to NBW (Fig. S1F). SELENOF knockout aggravates high-fat diet-induced obesity in mice, and disrupts glucose and lipid metabolism manifested in terms of hyperglycaemia, hepatic steatosis and elevated LDL-Cho [14]. Enhanced levels of cord blood Cer and associated reductions in placental selenoproteins may thus orchestrate dysregulated insulin signalling and lipid metabolic aberrations particularly in HBW. Pathways of ‘RHO GTPase cycle’ and ‘RHO GTPase effectors’ were also significantly correlated with changes in cord blood Cer levels of both LBW and HBW groups (Fig. 1D). In addition, ‘MHC class II antigen presentation’ was distinctly associated with elevated cord blood Cer in the LBW group. VLC-Cer were negatively associated with the protein level of spectrin beta-chain (SPTBN2), but positively associated with protein levels of human leukocyte antigen gene complex (HLA) class II histocompatibility antigen DP alpha 1 chain and DR beta 5 chain (HLA-DPA1, HLA-DRB5), as well as tubulin beta-6 chain (TUBB6) (Fig. 1E). SPTBN2 is a constituent of the microtubule machinery that translocates peptide-loaded HLA complexes to the surface of antigen presenting cells (APCs) for subsequent recognition by immune cells. HLA complexes bind extracellular peptides taken up via endocytosis and present these peptides onto the surface of APCs. The high cord blood Cer level therefore possibly compromises MHC class II antigen presentation in LBW. Increases in protein levels of the HLA complex subunits might represent compensatory mechanisms to cope with diminished levels of SPTBN2 and abated antigen presentation in the LBW group.

We examined whether cord blood Cer levels at birth could effectively predict metabolic status of offspring later in life. A separate cohort of 50 mother-infant dyads from the HAPO Study in Hong Kong was used to investigate if cord blood lipids measured at baseline could predict childhood metabolic status at the 7th year. Metabolic indices collected at the 7th year included BMI, waist circumference, skinfold thickness, fasting glucose, homeostatic model assessment for insulin resistance (HOMA-IR), total cholesterol (TC), high-density-lipoprotein-cholesterol (HDL-Cho), low-density-lipoprotein-cholesterol (LDL-Cho), triglyceride (TG) level, insulinogenic (INS) index, quantitative insulin sensitivity check index (QUICKI) and the oral disposition index (ODI) (Fig. 1F). Spearman correlation analyses demonstrated that several Cer species were positively correlated with TC, HDL-Cho and LDL-Cho measured at the 7th year. We then segregated the offspring into tertiles based on levels of LDL-Cho at the 7th year (Fig. 1G). The ROC curves presented satisfactory AUCs in discriminating offspring from discrete LDL-Cho tertiles for both training (AUC >0.9) and validation (AUC >0.8) sets (Fig. 1H). To evaluate the contribution of individual variables on overall predictive performance, one variable was removed consecutively and the same cross-validation procedures were applied, and the resultant changes in AUCs of the validation samples (10%×10) for discriminating offspring belonging to the 2nd tertile from the 3rd tertile of LDL-Cho at the 7th year were plotted (Fig. 1H). Notably, removing Cer d18:0/20:0 and Cer d18:1/20:0 from the predictive panel reduces AUCs by 0.009 and 0.018, respectively (Fig. 1I). Furthermore, the levels of Cer d18:0/20:0 and Cer d18:1/20:0 in baseline cord blood samples exhibited progressive increases across tertiles of LDL-Cho at the 7th year (Fig. 1J). We demonstrated the predictive potential of baseline cord blood Cer for childhood metabolic health defined in terms of elevated LDL-Cho. Corroborating our findings, the Cer content of aggregated LDLs in atherosclerotic lesions was found to be 10–50-fold higher than that of circulating LDLs, implying that Cer may promote LDL aggregation and plaque formation [15].

To conclude, our multi-omics investigation systematically reports cord blood sphingolipids functionally associated with changes in birth weight, and underscores the biological relevance of cord blood Cer in modulating intrauterine fetal growth and development. Our integrated analyses propose possible molecular pathways that orchestrate changes in fetal growth and metabolism triggered by aberrantly high levels of Cer within the intrauterine environment. The predictive potential of cord blood Cer toward childhood metabolic health was also demonstrated, thus supporting the crucial role of functional lipids within the cord blood in modulating fetal growth and in altering long-term metabolic health. Cord blood-based prediction facilitates early risk identification and provides an intervention window to minimize adverse life-long metabolic outcomes. As the endogenous levels of cord blood lipids are amenable to extrinsic interventions, our results present a new clinical dimension to monitoring and possibly fine-tuning intrauterine fetal growth and development to maximize the metabolic health benefits for both mother and child.

## Supplementary Material

nwae352_Supplemental_File
