# Supplementary material for: Cord blood ceramides facilitate early risk identification into childhood metabolic health
Source: Natl Sci Rev. 2024 Oct 3;11(10):nwae352. doi: 10.1093/nsr/nwae352 (PMC11519047; doi:10.1093/nsr/nwae352)
Supplement: nwae352_Supplemental_File [file nwae352_supplemental_file.docx]

**Supplementary Materials**

# Supplementary Method 1. Study design and patients

152 mother-infant dyads were consecutively enrolled in the Department of Gynaecology and Obstetrics at Peking Union Medical College Hospital, which included 69 neonates with NBW, 56 neonates with LBW, and 27 neonates with HBW. The cut-off value for low birth weight was set at 3000 g based on our previous finding that birth weight of < 3000 g is an independent risk factor of impaired glucose regulation, T2D, and metabolic syndrome later in life [1, 2]. Supplementary Table 1 summarizes the clinical characteristics of the recruited study participants. The study design and categorization of the recruited participants is shown in Figure 1. Written informed consent was obtained from all mothers participating in this study. The study was approved by the Ethics Committee of the Institutional Review Board at Peking Union Medical College Hospital (No. S-002). Recruitment and analysis of the Hong Kong cohort was approved by the Joint Chinese University of Hong Kong-New Territories East Cluster Joint Clinical Research Ethics Committee.

# Supplementary Method 2. Study participants of prospective cohort

A subset of 50 mother-infant dyads from the Hyperglycemia and Adverse Pregnancy Outcome (HAPO) Study Hong Kong subcohort [3] was leveraged to elucidate the predictive potential of functional cord blood lipids measured at baseline on offspring metabolism at 7^th^ year. The offspring attended a follow-up assessment at ~7-years of age, with information on sex, age, weight, height, skinfold thickness, and waist circumference information collected. All children underwent an oral glucose tolerance test with a glucose load of 1.75g/kg body weight (maximum 75g). Biochemical indices including plasma fasting glucose, insulin, triglyceride level, total cholesterol, high-density lipoprotein-cholesterol (HDL-Cho) and low-density lipoprotein-cholesterol (LDL-Cho) were also measured. Homeostatic model assessment for insulin resistance (HOMA-IR), insulinogenic index (IGI), quantitative insulin sensitivity check index [QUICKI; 1/ (log (Fasting Insulin) + log (Fasting Glucose)], and oral disposition index (ODI; HOMA-IS × insulinogenic index) were calculated to evaluate insulin sensitivity and insulin resistance.

**Supplementary Method 3. Plasma and placenta collection**

Venous blood of mothers was collected in EDTA tubes and venous umbilical cord blood specimens were collected immediately after the delivery of the baby. All specimens obtained were kept on ice and centrifuged at 4000 x g for 10 min following collection. The supernatant was collected and plasma aliquots were stored at -80°C until further biochemical assays. Placental tissues were collected by trained pathology personnel according to our previous protocol [4]. Following standard obstetrical practice, the placenta was collected in a clean sterile container. Three 1cm × 1cm × 1cm cuboidal sections were circumferentially excised from separate areas of the placenta, each located 4 cm from the cord insertion site by trained personnel. The placenta tissues were snap frozen in liquid nitrogen, and stored at -80°C for further analysis.

**Supplementary Method 4. Biochemical assays**

Glucose level was measured using the glucose oxidase method by an automated analyzer, while insulin was measured using a Human Insulin-Specific radioimmunoassay (RIA) kit (HI-14K, Millipore, MA, USA). The inter- and intra-assay coefficients of variations were 2.9–6.0% and 2.2–4.4%, respectively. Insulin-like growth factor 1 (IGF-1), a crucial hormone for fetal growth and development, was measured using a Human Insulin-Specific RIA kit (A15729, Beckman-Coulter, CA, USA). Leptin level was measured using a human leptin RIA kit (HL-81HK, Millipore, MA, USA). The inter- and intra-assay coefficients of variations for the leptin assays were 3.6–6.2% and 3.4–8.3%, respectively. High molecular weight-adiponectin (HMW-APN) concentration was determined using a quantitative ELISA (enzyme-linked immunosorbent assay) kit [DHWAD0, R&D Systems, Minneapolis, MN]. The intra- and inter-assay coefficients of variations were 2.6-3.7% and 8.3-8.6%, respectively. All samples were run in duplicates and in a blinded manner, as reported previously [5]. Samples in Hong Kong were processed as previously described [3].

**Supplementary Method 5. Quantitative lipidomics**

Lipids were extracted from 50 μL of maternal or cord blood samples using a modified Bligh and Dyer’s extraction protocol (double extraction) [6]. Briefly, 750 µL of chloroform: methanol: MilliQ H_2_O (3:6:1) (v/v/v) was added to plasma/cord blood samples and incubated at 1500 rpm for 1h at 4℃. At the end of the incubation, 350 µL of deionized water and 250 µL of chloroform were added to induce phase separation. The samples were then centrifuged and the lower organic phase containing lipids was extracted into a clean tube. Lipid extraction was repeated once by adding 450 µL of chloroform to the remaining aqueous phase, and the lipid extracts were pooled into a single tube. Organic extracts were dried in SpeedVac under OH mode and the lipid pellets were resuspended in chloroform: methanol (1:1, v/v) prior to mass spectrometric analyses. Targeted lipidomics was conducted on an Exion UPLC coupled to QTRAP 6500 Plus (Sciex) as we previously described [7, 8]. Separation of individual lipid classes of polar lipids by normal phase (NP)-HPLC was carried out using a TUP-HB silica column (i.d. 150x2.1 mm, 3 µm) with the following conditions: mobile phase A (chloroform: methanol:ammonium hydroxide, 89.5:10:0.5) and mobile phase B (chloroform:methanol:ammonium hydroxide:water, 55:39:0.5:5.5). Multiple reaction monitoring (MRM) transitions were set up for comparative analysis of various lipids. Individual lipids were quantified relative to their respective internal standards, including SM-d18:1/12:0, d_31_-SM-16:0/18:1, Cer d18:1/d_7_-15:0, glucosylceramide (GluCer) d18:1/16:0, obtained from Avanti Polar Lipids; d_3_-ganglioside (GM3) d18:1/18:0 and d_3_-lactosylceramide (LacCer)-d18:1/16:0 from Matreya LLC. Quantitated lipid levels were expressed in mol/L plasma or cord blood for statistical analyses [7-9].

**Supplementary Method 6. Protein extraction and proteomic analysis**

Placenta tissues were grounded in liquid nitrogen into fine powder, following which four volumes of lysis buffer (1% Triton X-100 with 1% protease inhibitor cocktail) were added, and the samples were sonicated on ice using a high-intensity ultrasonic processor (Scientz). The samples were then centrifuged at 12, 000 g at 4 °C for 10 min and the supernatant was collected and total protein concentration determined using the bicinchoninic acid (BCA) kit according to manufacturer’s protocol. The samples were reconstituted to a final concentration of 20% (m/v) trichloroacetic acid (TCA) for protein precipitation, vortexed and incubated for 2 h at 4 °C. The precipitate was collected by centrifugation at 4500 g for 5 min at 4 °C, and washed with pre-cooled acetone for 3 times then dried for 1 min. The protein samples were resuspended in 200 mM tetraethylammonium bromide (TEAB) and sonicated. Trypsin was then added at 1:50 trypsin-to-protein mass ratio for overnight digestion. Samples were reduced with 5 mM dithiothreitol (DTT) for 60 min at 37 °C then alkylated with 11 mM iodoacetamide for 45 min at room temperature in darkness. The peptides were desalted by Strata X SPE columns prior to LC-MS/MS. Tryptic peptides were resuspended in mobile phase A (2% acetonitrile in water containing 0.1% formic acid) and loaded onto a C18 analytical column (100 µm i.d. x 25 cm). Peptides were separated on a gradient at a flow rate of 500 nL/min, with mobile phase B (90% acetonitrile in water containing 0.1% formic acid) increasing from 6% to 22% over 36 min, which was further increased to 32% B in 16 min and finally to 80% B over 4 min. The gradient was maintained at 80% B for another 4 min. Eluted peptides were analysed on a Q Exactive^TM^ HF-X (ThermoFisher Scientific) with a nano-electrospray ion source (Spray voltage 2.1 kV, full MS scan resolution 120,000, scan range: m/z 350-1600). Up to 20 most abundant precursors were selected for MS/MS with 30 s dynamic exclusion, with higher energy collisional dissociation (HCD) fragmentation performed a normalized collision energy of 28%. Fragments were detected in the Orbitrap at mass resolution 45,000. The fixed first mass was set as 100 m/z; automatic gain control target was at 1e5 with an intensity threshold of 8.3e4 and a maximum injection time of 60 ms. MS/MS data were processed using the Proteome Discoverer search engine (v2.4.1.15). Tandem mass spectra were searched against the human SwissProt database (20395 entries) concatenated with reverse decoy and contaminants database. Mass error was set at 10 ppm for precursor ions and 0.02 Da for fragment ions. TMT-16 plex quantification was performed, and false discovery rate (FDR) of protein, peptide and propensity score matching (PSM) were adjusted to <1 %. Relative abundances of individual proteins were calculated by the intensity median of their corresponding unique peptides.

# Supplementary Method 7. Statistical analysis

# Clinical and biochemical characteristics were compared using ANOVA and chi-square test for continuous and categorical variables, respectively (Supplementary Table 1). A limma model was constructed to evaluate cord blood lipid changes associated with deviations in birth weights from the optimal NBW group. False discovery rate (FDR) was obtained using the Benjamini-Hochberg method. Differential lipids for LBW relative to NBW (LBW-NBW) and HBW relative to NBW (HBW-NBW) were summarized in forest plots (Figure 1A, Supplementary Figure 1A) with log_2_ fold-changes, P-value and FDR presented. Three notable groups of birth weight-associated lipids were boxed and their respective patterns of changes illustrated as line-plots of z-scores (Figure 1B, Supplementary Figure 1B). Protein levels of placental ceramide synthase 2 were compared between the three different birth weights using pairwise comparison results from Dunn’s tests (Supplementary Figure 1C). Significance was indicated using letter-based representation. Distribution of Cer d18:0/24:1 between cord blood and maternal blood were examined using Spearman correlation (Supplementary Figure 1D). Correlations between cord blood lipids and indices related to mothers (maternal indices) and fetal microenvironment (cord blood biochemistry) were examined using Spearman correlations (Figure 1C, Supplementary Figure 1E). Only statistically significant correlations (P<0.05) were illustrated, with sizes of dots indicating the magnitude of the P-values, and different colours denoting the directions of correlations (red: positive correlations, blue: negative correlations). Trans-omics integration of cord blood lipidomics and placenta proteomics data was conducted by calculating the Spearman correlations between placental proteins and cord blood lipids that were significantly associated with birth weight. Due to non-linear changes in the levels of cord blood lipids with deviations from the optimal birth weight group (NBW), correlation analyses were separately performed amongst the LBW+NBW and NBW+HBW participants. Over-representation analysis (ORA) of pathways was conducted based on significantly correlated proteins (P<0.05, |r|>0.7) against the Reactome database (Figure 1D). A network was built based on the proteins within the pathway and their correlated lipids. Significant pathways (P<0.05) were then ranked based on the number of connections in the largest sub-network (m). Density of each pathway was calculated by m/n, where n denotes the highest theoretically possible number of connections in the largest sub-network i.e. m*(m-1)/2, and represented by the length of individual scale bars. Lipid-protein correlation networks for individual enriched pathways were constructed using Spearman correlation (P<0.05), with red edges denoting positive correlations and blue edges indicating negative correlations (Figure 1E). The numbers on individual edges denote the magnitudes of the correlation coefficients of protein-lipid pairs. For the prediction of offspring childhood metabolism (Figure 1G), metabolic indices were collected at the 7^th^ year included BMI, waist circumference, skinfold thickness, fasting glucose, homeostatic model assessment for insulin resistance (HOMA-IR), total cholesterol, high-density-lipoprotein-cholesterol (HDL-Cho), low-density-lipoprotein-cholesterol (LDL-Cho), triglyceride level, insulinogenic index, quantitative insulin sensitivity check index (QUICKI) and the oral disposition index (ODI) (Figure 1F). Spearman correlation analysis was first conducted to uncover metabolic indices significantly correlated with the functional lipids measured in baseline cord blood samples (Figure 1F). Offspring were categorized into tertiles based on levels of LDL-Cho at 7^th^ year (Figure 1G). A lasso regression model was constructed using baseline cord blood lipid levels and baseline offspring clinical and metabolic indices to select variables significantly associated with LDL-Cho at 7^th^ year. For individual pairwise comparisons of groups by tertiles, linear regression-based receiver operating characteristic (ROC) curves with ten-fold cross validation was conducted using the lasso-selected variables. ROC curves were drawn based on the combined training samples and validation samples from the ten-fold cross validation (training: 90%x10 and validation: 10%x10) (Figure 1H). To evaluate the contribution of individual variables to overall predictive performance, variables were removed one at a time and the same cross-validation procedures were run, and the resultant changes in area under curve (AUC) of the validation samples (10%x10) for differentiating 3^rd^ tertile from 2^nd^ tertile of LDL-Cho at 7^th^ year were plotted (Figure 1I). Boxplots were generated to depict the levels of Cer d18:1/20:0 and Cer d18:0/20:0 in cord blood across the three tertiles. Significance was calculated by performing a t-test for the regression slope of linear regression model (Figure 1J).

**Supplementary Table 1. Baseline characteristics of study participants.**

Comparisons amongst the three groups were performed using one-way ANOVA for continuous variables and Pearson’s Chi-square test was used for categorical variables. Statistical significance was indicated using letter-based representations of all pairwise comparisons, such that two groups sharing a common letter were not significantly different at P< 0.05. Data were presented as mean (standard deviation), n (%). LBW: low birth weight; NBW: normal birth weight; HBW: high birth weight; BMI: body mass index; IGF-1: insulin-like growth factor 1; APN: adiponectin

| **Characteristic** | **LBW** | **NBW** | **HBW** | **P value** |
| --- | --- | --- | --- | --- |
| Sample size (n) | 56 | 69 | 27 |  |
| **Mothers** | | | | |
| Age (years) | 30.86 (3.70)^a^ | 31.46 (3.03)^a^ | 30.44 (3.14)^a^ | 0.341 |
| Pre-pregnancy weight (kg) | 54.17 (7.83)^b^ | 57.37 (9.25)^a^ | 61.48 (7.38)^c^ | 0.002 |
| Pre-pregnancy BMI (kg/m^2^) | 20.78 (2.78)^a^ | 21.40 (3.01)^a^ | 23.26 (2.54)^b^ | 0.002 |
| Gestational weight gain (g) | 15.62 (4.41)^a^ | 16.33 (4.44)^a^ | 19.85 (4.49)^b^ | 0.001 |
| Gestational BMI increase (kg/m^2^) | 6.00 (1.70)^a^ | 6.08 (1.58)^a^ | 6.94 (2.62)^a^ | 0.082 |
| **Umbilical cord blood parameters** | | | | |
| Insulin (uIU/ml) | 12.60 (11.54)^a^ | 13.38 (5.67)^a^ | 20.90 (12.09)^b^ | 0.001 |
| IGF-1 (ng/ml) | 45.40 (25.79)^a^ | 51.78 (16.61)^a^ | 91.40 (42.19)^b^ | <0.001 |
| Glucose (mg/dl) | 75.72 (18.98)^b^ | 85.22 (17.71)^a^ | 45.80 (13.54)^c^ | <0.001 |
| Leptin (ng/ml) | 6.77 (4.84)^b^ | 10.32 (6.46)^a^ | 15.24 (7.54)^c^ | <0.001 |
| APN (μg/ml) | 16.49 (7.69)^b^ | 11.69 (6.07)^a^ | 15.60 (5.86)^b^ | <0.001 |


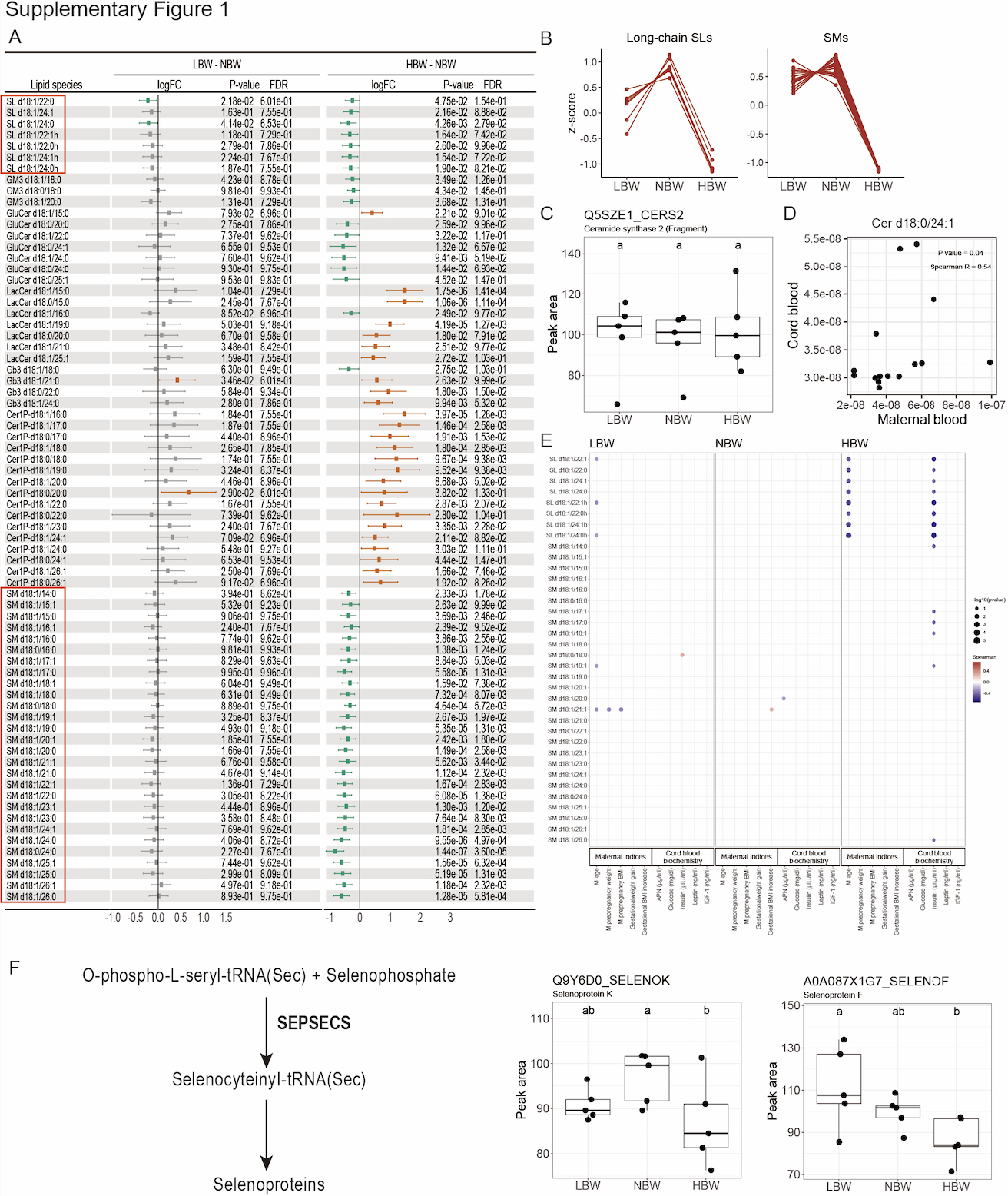


**Supplementary Figure 1. Cord blood sphingolipids.** (A) Functional cord blood sphingolipids associated with birth weights**.** Forest plot illustrating differential lipids for LBW relative to NBW (LBW-NBW) and HBW relative to NBW (HBW-NBW) with log2 fold change (95% CI), P value, and false discovery rate (FDR, 0.05; Benjamini-Hochberg method) presented. A limma model was used to evaluate the changes in cord blood ceramides associated with alterations in birth weight from the NBW group. Individual lipids were grouped according to lipid classes. Red rectangles denote lipids boxed up for emphasis. Green and red dots indicate significantly downregulated and upregulated lipids with reference to NBW group, respectively. **B)** Two notable groups of birth weight-associated sphingolipids and their respective patterns of changes illustrated as line plots drawn using z-scores. Long-chain SLs showed an inverted V pattern and SMs showed partial inverted V pattern. Y-axis is Z-score. **(C)** Protein Levels of placental ceramide synthase 2 among the different birth weights. Pairwise comparison results from Dunn’s tests were performed and indicated as letter-based representation, such that two groups sharing a common letter are not significantly different at P<0.05. **(D)** Scatterplot of infant cord blood ceramides compared with maternal cord blood ceramides. Distribution was examined using Spearman correlation. **(E)** Correlation between cord blood sphingolipids and clinical indices. Bubble plots illustrate the Spearman correlations between cord blood lipids and maternal indices and indices relevant to cord blood biochemistry in LBW, NBW and HBW. Only statistically significant correlations are shown; size of the bubble represents magnitude of P values from Spearman correlations, with positive correlations in red and negative correlations in blue and color intensity depicting the strength of the correlation. **(F)** Seleno amino acid metabolism pathway associated with birth weights. SEPSECS is a key enzyme in the synthesis of selenoproteins, including SELENOF and SELENOK; Changes of SELENOF and SELENOK expression in LBW, NBW and HBW groups; Statistical significance was represented using letter-based representation, with two groups sharing a common letter indicating no statistical significance between them. P-values presented were from Dunn’s test. NBW, normal birth weight; LBW, low birth weight; HBW, high birth weight; SL, sulfatides; Cer, ceramide; SM, sphingomyelin; APN, adiponectin; IGF-1, insulin-like growth factor 1; BMI, body mass index.

**Supplementary references**

1. Xiao, X., et al., *Evidence of a relationship between infant birth weight and later diabetes and impaired glucose regulation in a Chinese population.* Diabetes Care, 2008. **31**(3): p. 483-7.

2. Xiao, X., et al., *Low birth weight is associated with components of the metabolic syndrome.* Metabolism, 2010. **59**(9): p. 1282-6.

3. Tam, W.H., et al., *In Utero Exposure to Maternal Hyperglycemia Increases Childhood Cardiometabolic Risk in Offspring.* Diabetes Care, 2017. **40**(5): p. 679-686.

4. Zheng, J., et al., *The Placental Microbiome Varies in Association with Low Birth Weight in Full-Term Neonates.* Nutrients, 2015. **7**(8): p. 6924-37.

5. Zheng, J., et al., *Correlation of high-molecular-weight adiponectin and leptin concentrations with anthropometric parameters and insulin sensitivity in newborns.* Int J Endocrinol, 2014. **2014**: p. 435376.

6. Lam, S.M., et al., *A multi-omics investigation of the composition and function of extracellular vesicles along the temporal trajectory of COVID-19.* Nat Metab, 2021. **3**(7): p. 909-922.

7. Song, J.W., et al., *Omics-Driven Systems Interrogation of Metabolic Dysregulation in COVID-19 Pathogenesis.* Cell Metab, 2020. **32**(2): p. 188-202.e5.

8. Wang, X., et al., *Localized increases in CEPT1 and ATGL elevate plasmalogen phosphatidylcholines in HDLs contributing to atheroprotective lipid profiles in hyperglycemic GCK-MODY.* Redox Biol, 2021. **40**: p. 101855.

9. Lam, S.M., et al., *Extensive characterization of human tear fluid collected using different techniques unravels the presence of novel lipid amphiphiles.* J Lipid Res, 2014. **55**(2): p. 289-98.
